# Supplementary material for: Impact of the presence of a prosthetic implant and transition to oral stepdown therapy on relapse rates and mortality in uncomplicated Staphylococcus aureus bacteremia treated with 14 days of antibiotics: a retrospective cohort study
Source: Microbiol Spectr. 2025 May 23;13(7):e03337-24. doi: 10.1128/spectrum.03337-24 (PMC12211085; doi:10.1128/spectrum.03337-24)

**Table S1.** Diagnostic workup for SAB

|  | **Prosthetic implant n=58** | **Intravascular or cardiac prosthesis***  **n=42** | **No prosthesis n=130** | **p-value**** |
| --- | --- | --- | --- | --- |
| TTE | 53 (91%) | 38 (90%) | 120 (92%) | 1 |
| TEE | 17 (30%) | 14 (33%) | 18 (14%) | **0.05** |
| Venous doppler | 16 (28%) | 10 (24%) | 53 (41%) | 0.27 |
| FDG-PET/CT | 13 (22%) | 12 (29%) | 5 (4%) | **0.001** |
| TAP CT | 17 (30%) | 13 (31%) | 30 (23%) | 0.49 |
| Cardiac CT | 4 (7%) | 4 (10%) | 0 (0%) | **0.01** |
| CT: computed tomography; FDG-PET: fluorodeoxyglucose positron emission tomography; TAP: thoraco-abdominopelvic; TEE: transoesophageal echocardiography; TTE: transthoracic echocardiography. *Pacemaker (n=17), implanted defibrillator (n=8), prosthetic heart valve (n=14), vascular graft (n=15), HeartMate (n=1). **Comparison between Prosthetic implant and No prosthesis. | | | | |

**Table S2.** Antibiotic treatment and follow-up blood culture

|  | **Prosthetic implant n=58** | **No prosthesis  n=130** | **p-value** |
| --- | --- | --- | --- |
| **Antibiotic treatment** |  |  |  |
| **Initial IV antibiotic** |  |  |  |
| Cefazolin (CFZ) or Cloxacillin (CLO) | 37 (64%) | 75 (58%) | 0.70 |
| Vancomycin | 19 (33%) | 44 (34%) | 1 |
| MSSA | 17/19 (89%) | 31/44 (70%) | 0.68 |
| Switch to CFZ/CLO when MSSA | 15/17 (88%) | 27/31 (87%) | 1 |
| Amoxicillin | 1 (2%) | 1 (1%) | 0.53 |
| Others^ß^ | 1 (2%) | 10 (8%) | 0.18 |
|  |  |  |  |
| **Switch to oral route** | 28 (48%) | 80 (61%) | 0.43 |
| Days from antibiotic start, median (IQR) | 7 (5 - 9) | 7 (5 - 9) | 0.47 |
| Clindamycin | 14 (50%) | 36 (45%) | 0.85 |
| Cotrimoxazole | 6 (21%) | 27 (34%) | 0.49 |
| Amoxicillin | 2 (7%) | 9 (11%) | 0.73 |
| Levofloxacin or ciprofloxacin | 1 (4%) | 6 (7%) | 0.68 |
| Others^∂^ | 4 (14%) | 1 (1%) | **0.02** |
| **Duration of antibiotic therapy: days, median (IQR)** | 14 (14-16) | 14 (14-15) | **0.02** |
|  |  |  |  |
| **Persistent bacteremia* ≥ 72 hours, n (%)** | 11 (19%) | 10 (8%) | **0.05** |
|  |  |  |  |
| IQR: interquartile range; MSSA: methicillin-susceptible *Staphylococcus aureus*; SD: standard deviation. ^ß^Tecoplanin (n=5), piperacillin-tazobactam (n=3), meropenem (n=2) and cefepim (n=1). ^∂^Oral cloxacillin (n=2), amoxicillin-clavulanate (n=3). *Some patients were treated more than 14 days, because day 1 start at the first negative blood culture. **From antibiotic start to final positive blood culture. | | | |
|  |  |  |  |

**Table S3.** Simple logistic regression for prediction of 90-day SAB relapse

|  | **Univariate analysis** | |
| --- | --- | --- |
|  | OR (95% CI) | p value |
| **Prosthetic implant** | 7.0 (0.9 - 144) | 0.09 |
| **Oral stepdown therapy** | 0.7 (0.1 - 6.2) | 0.76 |
|  |  |  |
| Age | 1 (1 - 1.1) | 0.31 |
| SOFA ≥ 2 | 0.82 (0.04 - 6.6) | 0.87 |
| MRSA | -^∂^ | - |
| Blood culture TTP < 10 h | 2.3 (0.3 - 19) | 0.41 |
| SAB duration ≥ 72 hours* | 2.7 (0.1 - 23) | 0.39 |
|  |  |  |
| Primary infectious focus |  |  |
| Catheter-related | 0.2 (0.01 - 1.7) | 0.19 |
| Primary cutaneous | 3.8 (0.2 - 31) | 0.26 |
| Pneumonia | 4.8 (0.2 - 41) | 0.25 |
| Urinary tract infection | -^∂^ | - |
| Unknown | 2.5 (0.1 - 20) | 0.44 |
| Other** | -^∂^ | - |
| CI: confidence interval; MRSA: methicillin-resistant *Staphylococcus aureus*; OR: odds ratio; SAB: Staphylococcus aureus bacteremia; SOFA: sequential organ failure assessment; TTP: time to positivity. ^∂^: no relapse in this group, logistic regression not applicable; * From antibiotic start to final positive blood culture. **Pacemaker/defibrillator lodge infection (n=3), surgical site infection (n=2), biliary tract infection (n=2), peritonitis (n=1), parotiditis (n=1), diabetic foot infection (n=1). | | |
|  | | |

| **Patient** | **Time of relapse**** | **Prosthetic implant** | **Initial diagnosis**  **Primary focus** | **Initial workup** | **Duration of SAB (hours)** | **Antibiotic duration (days)** | **Oral stepdown therapy** | **2nd BSI workup** | **Final diagnosis** | **2nd treatment** | **Outcome at 90-day** |  |
| --- | --- | --- | --- | --- | --- | --- | --- | --- | --- | --- | --- | --- |
| 1 | 32 | none | Covid-VAP | TOE D5, TAP-CT | < 24h | 14 | No | TOE, TAP CT | Covid-VAP sphenoidal sinusitis | 14 days | alive |  |
| 2 | 8 | prosthetic valve vascular graft | Unknown (8 days after vascular graft implantation) | TOE D14 | 72h | 16 | No | TOE, FDG-PET/CT | unproven TAVI-IE | 6 weeks | alive |  |
| 3 | 8 | prosthetic valve pacemaker picc-line* | CRBSI with superficial occlusive thrombophlebitis | TTE, Doppler | 24h | 14 | Yes | TTE, TAP CT,  FDG-PET/CT | unproven PM-IE septic pulmonary embolism peritonitis (peritoneal dialysis) | 6 weeks | Unrelated death  (gastrointestinal bleeding) |  |
| 4 | 7 | vascular graft orthopaedic prosthesis | Cutaneous | TTE, AP-CT | < 24h | 14 | Yes | TOE, AP-CT,  FDG-PET/CT | infected psoas haematoma | 4 weeks | alive |  |
| CRBSI: catheter-related bloodstream infection; CT: computed tomography; FDG-PET: fluorodeoxyglucose positron emission tomography; picc-line: peripherally inserted central catheter; PM-IE: pacemaker infective endocarditis; TAP: thoracoabdominopelvic; TEE: transoesophageal echocardiography; TTE: transthoracic echocardiography. *Ablation on the same day the first positive blood culture was drawn. **From the end of antibiotic therapy to the first recurrent positive blood culture | | | | | | | | | | | | |

**Table S4.** Description of the four patients with SAB relapse

**Figure S1.** Survival curve


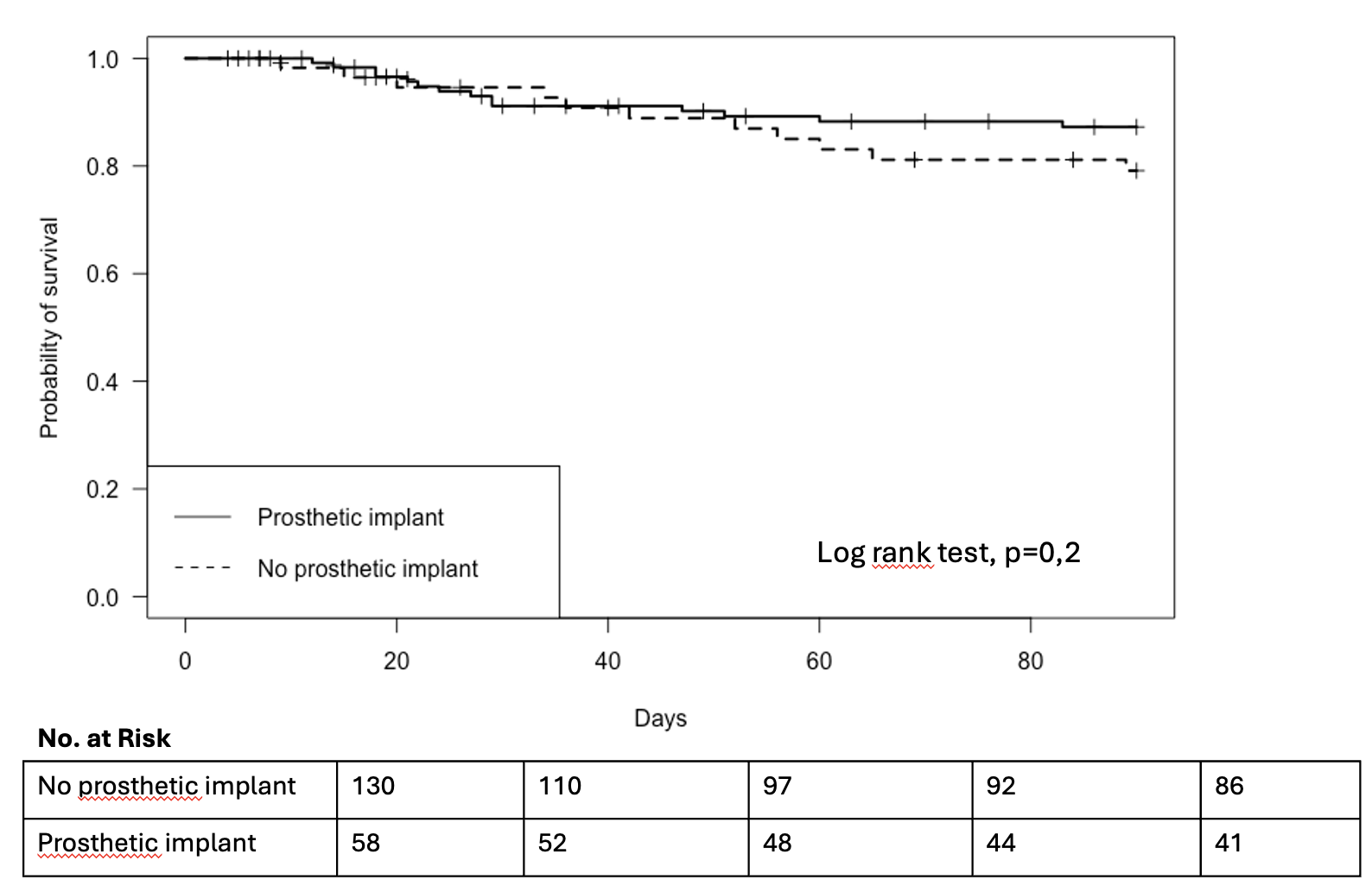

Supplement: Supplemental material — Tables S1 to S4; Fig. S1. [file spectrum.03337-24-s0001.docx]
